# The Effect of Ultrafine-Grained Microstructure on Creep Behaviour of 9% Cr Steel

**DOI:** 10.3390/ma11050787

**Published:** 2018-05-12

**Authors:** Petr Kral, Jiri Dvorak, Vaclav Sklenicka, Takahiro Masuda, Zenji Horita, Kveta Kucharova, Marie Kvapilova, Marie Svobodova

**Affiliations:** 1 Institute of Physics of Materials, Academy of Sciences of the Czech Republic, Zizkova 22, 616 62 Brno, Czech Republic; dvorak@ipm.cz (J.D.); vsklen@ipm.cz (V.S.); kucharova@ipm.cz (K.K.); kvapilova@ipm.cz (M.K.); 2 Department of Materials Science and Engineering, Faculty of Engineering, Kyushu University, Fukuoka 819-0395, Japan; masuda@zaiko6.zaiko.kyushu-u.ac.jp (T.M.); horita@zaiko.kyushu-u.ac.jp (Z.H.); 3 UJP PRAHA a.s., Nad Kamínkou 1345, 156 10 Praha-Zbraslav, Czech Republic; svobodova@ujp.cz

**Keywords:** creep-resistant 9%Cr steels, ultrafine grained materials, creep, electron back scatter diffraction

## Abstract

The effect of ultrafine-grained size on creep behaviour was investigated in P92 steel. Ultrafine-grained steel was prepared by one revolution of high-pressure torsion at room temperature. Creep tensile tests were performed at 873 K under the initially-applied stress range between 50 and 160 MPa. The microstructure was investigated using transmission electron microscopy and scanning electron microscopy equipped with an electron-back scatter detector. It was found that ultrafine-grained steel exhibits significantly faster minimum creep rates, and there was a decrease in the value of the stress exponent in comparison with coarse-grained P92 steel. Creep results also showed an abrupt decrease in the creep rate over time during the primary stage. The abrupt deceleration of the creep rate during the primary stage was shifted, with decreasing applied stress with longer creep times. The change in the decline of the creep rate during the primary stage was probably related to the enhanced precipitation of the Laves phase in the ultrafine-grained microstructure.

## 1. Introduction

Methods that cause severe plastic deformation (SPD) are very promising for the production of large volumes of ultrafine-grained (UFG) materials for the purpose of mechanical property testing [1,2,3,4,5]. At the present time, many SPD methods are known [1], such as high-pressure torsion (HPT) [6], equal-channel angular pressing [7], high pressure sliding [8], multiaxial forging [9] and accumulative roll bonding [10]. All of these SPD methods can be repeated several times in order to introduce a large plastic deformation into bulk materials. The main difference between various SPD methods is their effectivity in microstructure refinement. The most promising method in terms of effectivity is likely HPT, because UFG microstructure is already attained after 1 HPT revolution [1,11]. It has been shown that the HPT method can be repeated more than 1000 times without significant change in specimen dimensions [12], enabling the introduction of large strain into hard-to-deform materials at room temperature, such as metallic glasses or ceramics [13,14]. Thus, HPT is a very promising method for the processing of unique materials to be used for the fundamental study of features, such as phase transformations [12,15], amorphization of the crystalline phases [5] or deformation mechanisms influencing superplasticity [16]. 

SPD leads to the formation of unique microstructures that may contain a high dislocation density and varied proportions of low-angle boundaries (LAGBs) and high-angle boundaries (HAGBS) depending on the processing conditions [17,18,19]. Previous work has shown that SPD-processed materials may be unstable even at room temperature, and grains dynamically coarsen during creep testing at temperatures <0.3*T*_m_ [10,20,21]. Recent studies [22,23,24] have reported that the creep behaviour of SPD-processed materials is significantly influenced by the proportions of HAGBs and LAGBs. These properties can strengthen the material’s ability to act against dislocation glide or soften the material by processes associated with grain boundaries, such as grain boundary sliding or enhanced dislocation recovery.

Creep resistant 9%Cr steels containing a tempered martensitic microstructure were developed as high temperature components of power plants working at temperatures about 873 K [25,26]. Creep strength is influenced by the stability of the supersaturated solid solution, precipitates, martensite laths and subgrains [25,26,27,28,29,30]. The decrease in substitutional strengthening due to the formation of the Laves phase and the consumption of fine MX carbides pinning subgrains and dislocations by *Z*-phase formation leads to a large detrimental effect on the creep resistance. The Laves phase is an important intermetallic phase which significantly influences the microstructure stability and creep behaviour of P92 steel [31,32]. The Laves phase has a hexagonal structure and can be observed as Fe_2_W or (Fe,Cr)_2_(W,Mo). The dynamic precipitation of the Laves phase during creep is heterogeneous and occurs predominantly along boundaries and M_23_C_6_ particles [33,34].

It was found that the clustering of Laves phase particles at prior austenite grain boundaries may act as potential places for the nucleation of creep cavities [25,31]. The presence of cavities at grain boundaries can be associated with a concentration of high local stress induced by grain boundary sliding (GBS). Thus, grain boundaries are an important structure constituent influencing creep behaviour, especially in nano or UFG materials [35,36,37]. Our previous work [38] revealed that 1 HPT revolution at room temperature leads to the saturation of microstructure characteristics in UFG P92 steel at an equivalent strain higher than 20. It was shown that P92 steel with a UFG microstructure accelerates the formation of the Laves phase during creep in comparison with a coarse-grained state. The aim of the present work was to investigate creep behaviour at 873 K under different applied stresses and the role of the Laves phase in creep of UFG P92 steel. The results showed that the reduction of the grain size to the ultrafine-grained region using HPT deteriorated the creep resistance of UFG P92 steel in comparison with the coarse-grained (CG) state. The creep results in the present work also showed that the stress exponent, $n={\left({\delta \ln\dot{\varepsilon }}_{m}/\delta \ln\sigma \right)}_{T}$, determined for UFG P92 steel is significantly lower than the values published for CG state tested under the same creep conditions. The decrease in the value of the stress exponent, *n*, in the UFG state is related to the reduction of the grain size to the ultrafine-grained region. Thus, the experimental results were compared with modelled curves of creep mechanisms where grain size plays an important role.

## 2. Experimental Material and Procedures

A commercial P92 steel pipe made in Products Tubulares (Valle de Trapaga, Spain), s.a.u. with a 350 mm outer diameter and a 39 mm wall thickness was used in the present investigation. Its chemical composition is given in Table 1. The as-received P92 steel was normalized and tempered [39]. 

**Figure 1:**
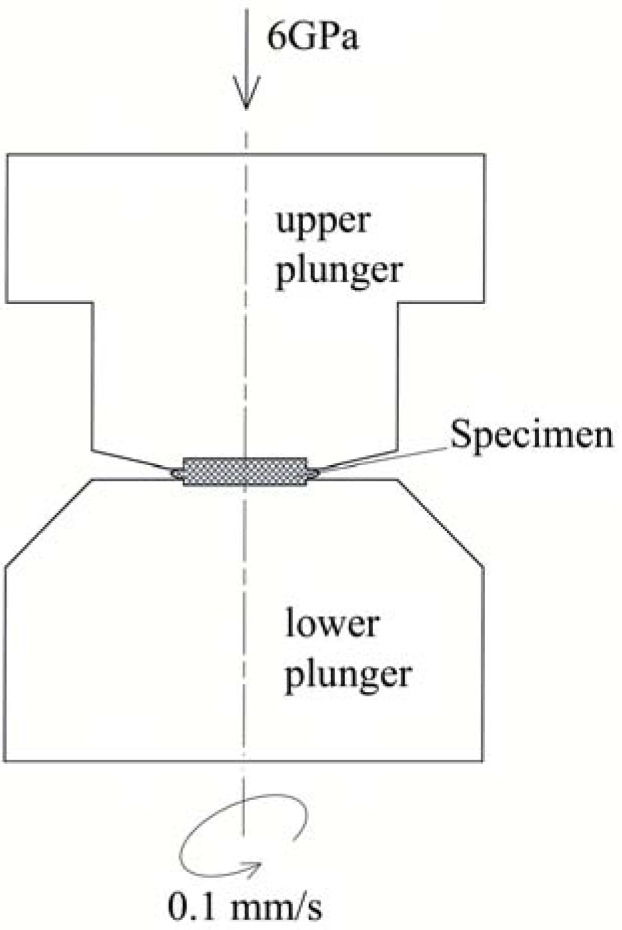
Schematic illustration of the high pressure torsion (HPT) process.

The ultrafine-grained microstructures were prepared by high pressure torsion (HPT). The 30 mm diameter discs with 1.1 mm thickness were made from the cylinder that was manufactured from the wall perpendicular to the longitudinal axis of the as-received pipe. Each of the discs was subjected to HPT processing (Figure 1) at room temperature by 1 revolution under a pressure of 6 GPa. The value of the von Mises equivalent strain by HPT was calculated according to the following equation:

(1) $$\varepsilon_{eq}=2\pi rN/\sqrt{3}t$$

where *r* is the distance from the torsion axis, *N* is the number of turns and *t* is the thickness of the disc. Tensile creep tests were carried out at 873 K under different initial applied stresses.

The creep testing was performed in a protective argon atmosphere using flat specimens with a 10 mm gauge length and a cross section of approximately 3 × 1 mm^2^. The gauge lengths of the creep specimens were extracted from the disc parts with an equivalent strain of about 20–30. The microstructure investigations were performed by means of scanning (SEM) and transmission electron microscopy (TEM). The TEM studies were carried out on carbon extraction replicas and thin foils using a JEOL 2100 F (JEOL Ltd., Tokyo, Japan) microscope operating at 200 kV. Electron backscatter diffraction (EBSD) analyses were performed using SEM TESCAN LYRA 3(Tescan, s.r.o., Brno, Czech Republic) equipped with a NordlysNano detector operating at an accelerating voltage of 20 kV with the specimen tilted at 70°. The microstructures were investigated with the metallographic section perpendicular to the torsion axis. The EBSD analyses were performed on 20 µm × 16 µm areas using a step size of 50 nm, and the data were analysed using HKL Channel 5 software(Oxford Instruments, High Wycombe, United Kingdom) developed by Oxford Instruments. The misorientation angle, θ = 15°, was chosen as the minimal angle for identification of HAGBs. The lowest limit of 2° was selected due to the angle resolution of EBSD as the minimal misorientation angle for LAGBs. The grain size was determined as the arithmetic mean of the results of measurements in two perpendicular (horizontal, vertical) directions.

## 3. Results

### Creep Behaviour

**Figure 2:**
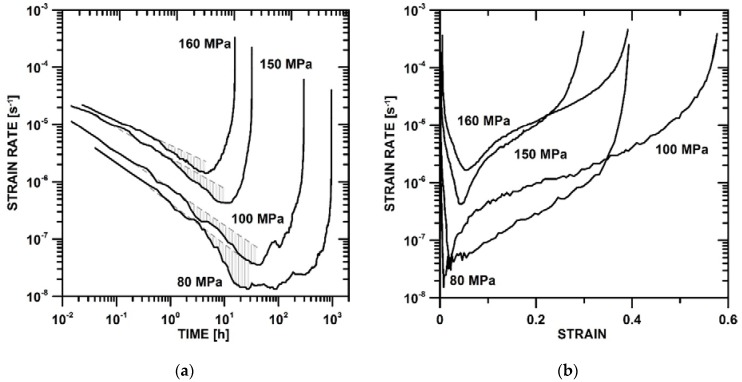
Creep curves for UFG P92 steel: (**a**) strain rate vs. time and (**b**) strain rate vs. strain.

The tensile creep curves of UFG P92 steel tested at 873 K under different initial stresses are shown in Figure 2a,b. The dependences of the creep rate against time (Figure 2a) demonstrate the change in the slopes of the creep curves in the primary stage. The change in the slope can be indicated by deviation of the experimental creep curves from dashed grey lines in Figure 2a. Region I is shown by a straight line and region II is characterized by strengthening during the primary creep stage (deviation from dashed grey line).

The slope in the different regions of the primary stage may be characterized by absolute values of $\left|\frac{d\dot{\varepsilon }}{dt}\right|$ (Table 2). The results demonstrate that the intensity of the decrease in the creep strain rate increases with a decreasing value of initial stress. One can see that the change in the slope is shifted with a decreasing value of initial stress with a longer creep time.

The curves of creep strain rate vs. strain exhibited a systematic decrease of strain to the minimum creep rate with a decreasing value of initial stress. It can be seen that the largest elongation of tensile specimens occurs during the tertiary stage (Figure 2b).

**Figure 3:**
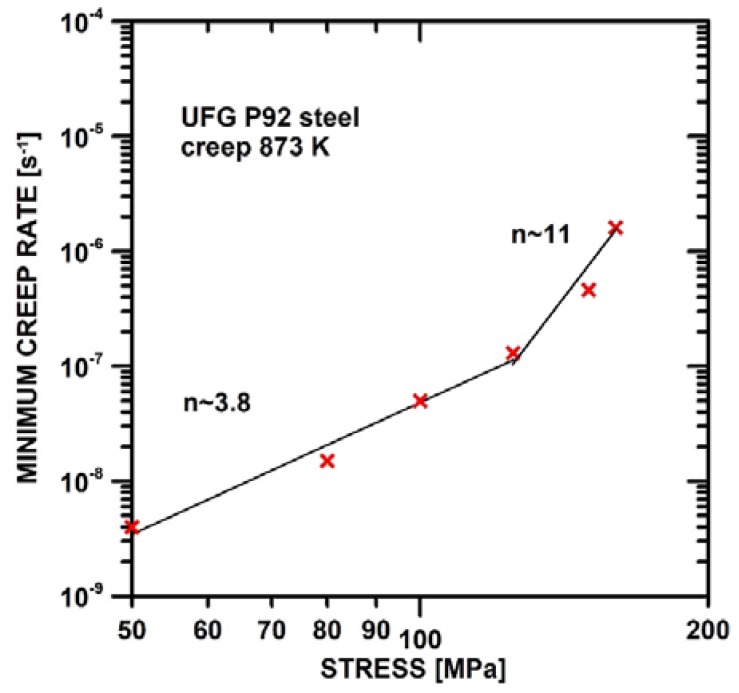
Stress dependence of the minimum creep rate measured for UFG P92 steel.

Figure 3 demonstrates the stress dependences of the minimum creep rate, ${\dot{\varepsilon }}_{m}$, for UFG P92 steel measured at 873 K. The stress exponent of the minimum creep rate, *n*, exhibits the value ~11 at stresses between 125 and 150 MPa. At lower applied stresses (<125 MPa), the value of stress exponent *n* decreases to *n* ~3.8. The decrease in the value of *n* with a decreasing applied initial stress may indicate a potential change in the operating deformation mechanism.

## 4. Microstructure Investigation

### 4.1. After HPT 

**Figure 4:**
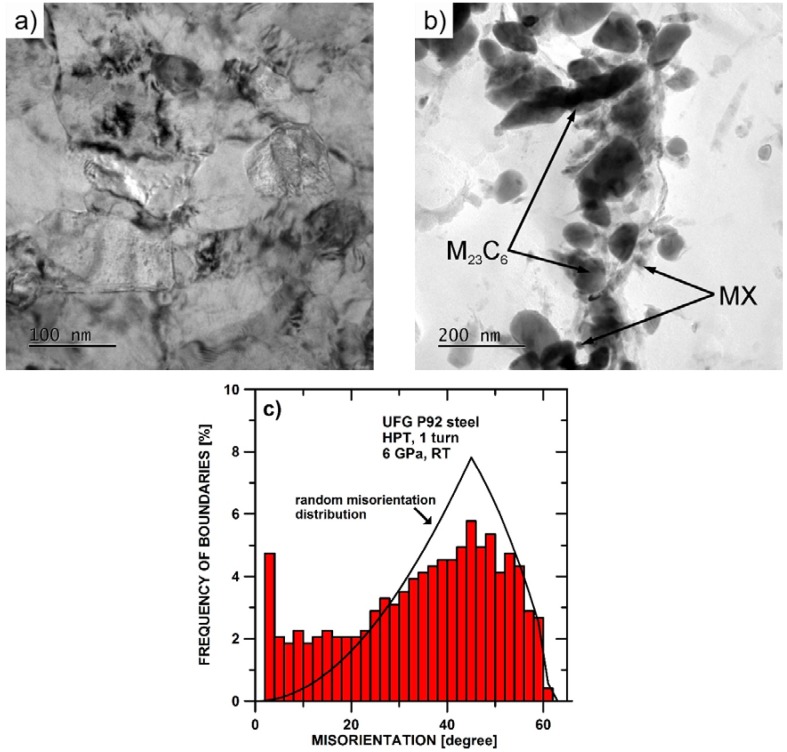
Microstructure of P92 steel processed by HPT during one revolution: (**a**) TEM micrograph of microstructure; (**b**) TEM micrograph of extracted precipitates and (**c**) distribution of misorientation angles.

The microstructure of P92 steel processed by HPT during 1 revolution at room temperature consisted of grains with a mean size of 0.14 μm (Figure 4a). The investigation of the precipitates revealed that the microstructure after the HPT processing contained M_23_C_6_ and MX carbides (Figure 4b). The analysis of the microstructure using EBSD showed that the grain boundaries exhibited nearly random distributions of misorientation angles (Figure 4c).

### 4.2. After Creep

**Figure 5:**
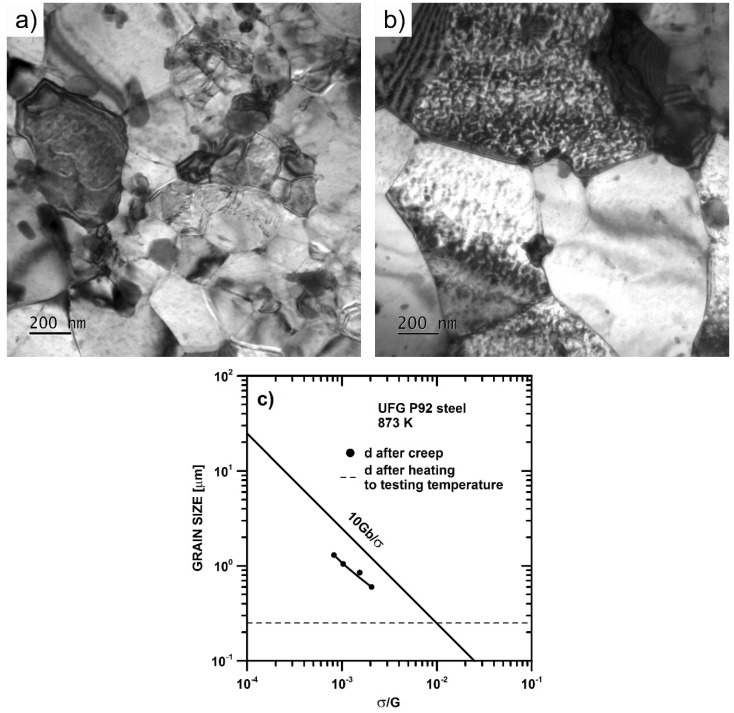
Microstructure of UFG P92 steel after creep testing: (**a**) the grip part of the specimen tested at 873 K and 150 MPa; (**b**) the gauge part of specimen tested at 873 K and 150 MPa and (**c**) coarsening of microstructure in gauge part against the normalized initial stress.

The microstructure after creep testing at 873 K and 150 MPa is shown in Figure 5. One can see that thermal exposure of the UFG microstructure in the grip part of the tensile specimen (time to fracture *t*_f_ ~30 h) tested at 873 K led to the coarsening of grains (Figure 5a).

Figure 5b demonstrates that creep deformation in the gauge length at 873 K caused additional coarsening of the grains, and the mean grain size after creep testing was ~0.85 μm. Figure 5c shows the evolution of mean grain size measured by EBSD with the normalized initial applied stress. It can be seen that the mean grain size after creep testing was finer than the steady-state subgrain size. The line in Figure 5c describing the steady-state subgrain size was determined by the following equation:

(2) $$w=k_{w}\frac{Gb}{\sigma },$$

where *b* = 2.48 × 10^−10^ m is the length of the Burgers vector, *G* is the shear modulus at a given temperature, *T*, and *k*_w_ was set equal to 10 [29]. The shear modulus, *G*, was determined using the temperature dependence of shear modulus: *G* = 97,400 − 0.039T [40].

**Figure 6:**
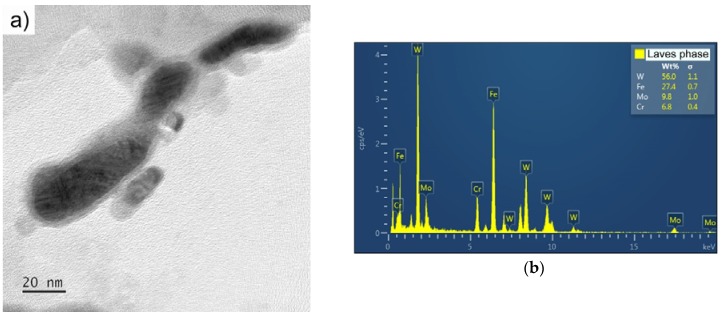
TEM micrograph of precipitates on extraction carbon replica in UFG P92 steel after creep testing at 873 K and 150 MPa: (**a**) Laves phase and (**b**) EDS spectrum of Laves phase.

**Figure 7:**
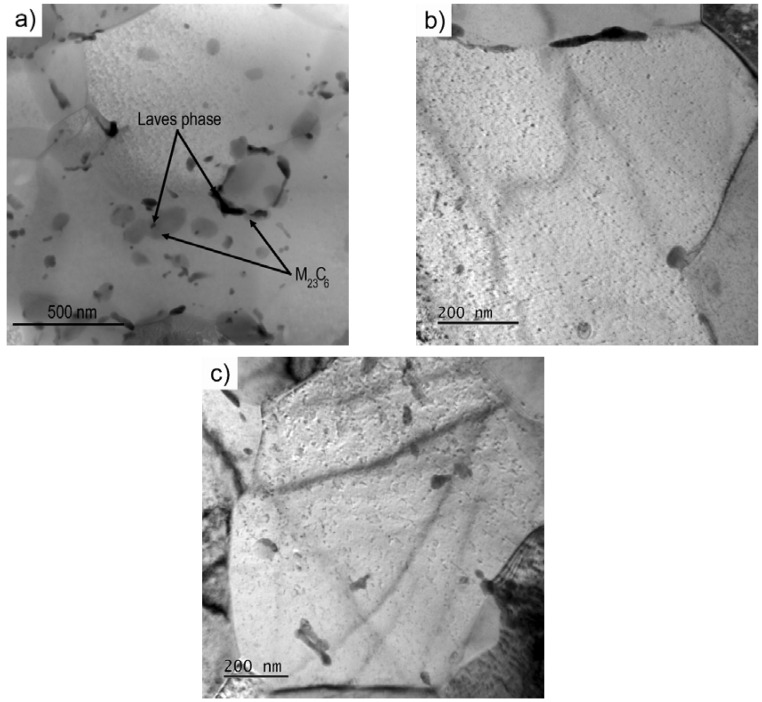
Distribution of precipitates in the gauge part of the specimen tested at 873 K and 150 MPa: (**a**) M_23_C_6_ carbides; (**b**) grain boundaries and (**c**) grain interior.

The investigation of the precipitates on the extraction carbon replicas revealed the formation of the Laves phase during creep testing (Figure 6). Microstructure images from SEM (Figure 7a) and TEM (Figure 7b,c) of UFG P92 after creep are shown in Figure 7. The images show the distribution of precipitates in the microstructure.

One can see that Laves phase was located at the M_23_C_6_ carbides (Figure 7a), grain boundaries (Figure 7b) and grain interiors (Figure 7c). Laves phase particles located at M_23_C_6_ carbides and grain boundaries were larger in comparison with particles formed in the interior of the grains. These results demonstrate that large particles of the Laves phase very often have an irregular shape and grow predominantly along the interfaces of M_23_C_6_ carbides and grain boundaries.

The distribution of the large particles of the Laves phase was heterogeneous and clusters formed predominantly at the matrix/M_23_C_6_ carbides interface and boundaries.

## 5. Discussion

In the present work, it was observed that the application of HPT at room temperature led to a significant reduction in grain size down to the ultrafine-grained region that is characterized by grain sizes ranging from 0.1 to 1 μm. Thus, the grain size in UFG P92 was comparable to the size of martensite lath and subgrains in P92 steel with standard coarse grain size [25,41,42]. The misorientation distribution measured in UFG P92 showed that the boundaries have a nearly random distribution of misorientation angles. However, a typical martensitic structure in P92 usually exhibits a bimodal distribution of boundaries, with the absence of boundaries between 21° and 47° [43].

The microstructure investigation showed that creep testing at 873 K caused coarsening of the microstructure, but the mean grain size was still close to the UFG region even after creep exposure for a duration of about 1000 h. The enhanced microstructure stability of UFG P92 steel at 873 K was influenced by the presence of M_23_C_6_ and MX carbides that retarded the movement of grain boundaries by their “pinning effect”. These carbides are formed during standard heat treatments, such as normalization and tempering. However, during creep exposure, the Laves phase can be formed in P92 steel. Previous work [33,44] has shown that the occurrence of the Laves phase in P92 steel during creep testing can have two opposite effects on the creep strength. The formation of the fine Laves phase enhanced the creep strength during the primary stage. Abe [44] reported that the improvement in creep strength is due to the dependence of strain rate vs. time, i.e., a sudden decrease in strain rate. Similar strengthening was also found in UFG P92 but it was shifted to shorter times and faster strain rates in comparison with coarse-grained steel [44]. It has been observed [45,46] that temperature and probably strain are important for the formation of the Laves phase. The present results suggest that primary creep strain plays an important role in Laves phase formation. When the primary creep strain decreases at lower stress levels, a longer time is needed for the formation of the Laves phase. In the present study, the Laves phase was formed at the boundaries and also in the interior of the grains. The formation of the Laves phase outside of M_23_C_6_ carbides led to a decrease in mean particle distance, and thus, an improvement in precipitation strengthening. The enhanced growth of the Laves phase along HAGBs (prior austenite grain boundaries and martensite lath) in comparison with LAGBs was observed in coarse-grained P92 steel [25,47]. This was caused by faster diffusion of solute elements along HAGBs as opposed to slower bulk diffusion and diffusion along dislocation cores. The present study also showed the formation of the Laves phase in the interior of the grains. This formation can be caused by fine Laves phase at boundaries of fine grains at the beginning of creep testing. After this, the HAGBs migrated due to temperature and time exposure and creep strain. Thus, the Laves phase originally formed at the boundary was displaced into the grain interior. The growth of Laves phase in the grain interior decelerated due to slower bulk diffusion in comparison with grain boundary diffusion. Similar observations were made for coarse-grained 9% Cr steel [48]. It was also observed that precipitates aligned along subgrain boundaries coarsened faster in comparison with precipitates that formed in the subgrain interior due to the migration of the subgrain boundary. However, the observation of the Laves phase in the interior may have been influenced by the presence and mutual interaction of mobile dislocations that can serve as nucleation sites for precipitation. Recent studies [49,50] have found that severe plastic deformation influences the precipitation kinetics and leads to the formation of finer particles in comparison to coarse-grained states due to the enhanced nucleation of precipitates at dislocation as well as dislocation tangles induced by SPD.

The creep results demonstrated that the strengthening occurring during creep testing improved creep strength, but only temporarily. This effect was also found in coarse-grained 9% Cr steel and was explained by fast coarsening of the Laves phase during continuation of creep exposure [44].

Because the Laves phase coarsened during continuous creep exposure and so its formation subsequently deteriorated the creep strength, mainly due to the increase in interparticle spacing and depletion of W from the solid solution.

**Figure 8:**
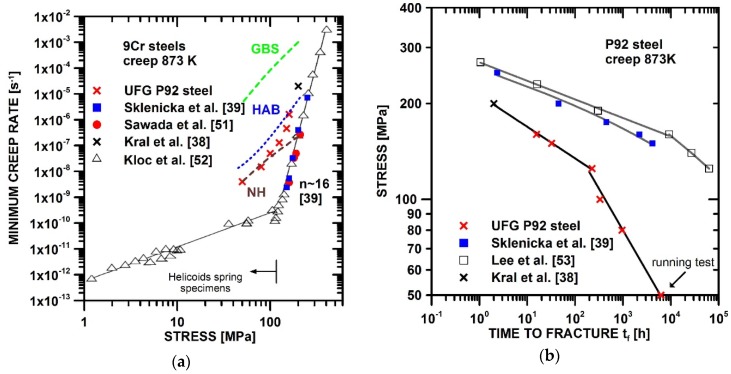
Comparison of the results measured in the present work with various creep models and results presented in other works [39,51,52,53]: (**a**) minimum creep rate vs. stress and (**b**) stress vs. time to fracture.

Figure 8 shows a comparison of the creep results measured in the present work with creep data published in other works [39,51,52,53] for coarse-grained 9% Cr steel. The comparison demonstrates that the transformation of tempered martensitic microstructure to a UFG microstructure led to the deterioration of creep resistance in comparison with coarse-grained P92 steel.

The value of the stress exponent of the minimum creep rate, *n*, measured in UFG P92 decreased significantly with a decreasing value of applied stress. The significant decrease in the stress exponent, *n*, with the reduction of applied stress (Figure 8a) has also been observed in coarse-grained 9% Cr steels [52]. Sklenicka et al. [39] showed that the value of the stress exponent, *n*, measured at 873 K is about 16 in CG P92 steel. The comparison of the values of the stress exponent, *n*, measured in UFG (Figure 3) and CG P92 steel (Figure 8a) demonstrates that the UFG state exhibits a lower value of the stress exponent, *n*, measured under the same conditions. The decrease in the stress exponent, *n*, in UFG state can be associated with the grain size reduction. It is generally accepted that in UFG materials, the grain-boundary mediated processes play an important role [22,23,24]. Among the creep processes significantly influenced by grain size are processes such as grain boundary sliding and migration, enhanced recovery of dislocations at HABGs and diffusion creep. The equation and material parameters used for the modelling of creep behaviour are shown in Appendix A. These equations use the dependence of grain size on the applied stress showed in Figure 5c; thus, the modelled curves are curved. 

The occurrence of superplastic behaviour in UFG materials characterized by *n* ~2 has often been observed for strain rates in the region of 10^−2^–10^−4^ s^−1^ [54,55]. It is generally accepted that the rotation of grains due to sliding along boundaries leads to the formation of random orientations (weakening of texture). However, Kral et al. [38] observed that the microstructure of UFG P92 steel after creep exhibited a relatively strong texture in the gauge length that was inherited from the HPT-processed state. The experimentally-measured data for UFG P92 steel were shifted to significantly slower strain rates in comparison with the GBS mechanism (Equation (A1)). The difference between experimentally-measured and modelled results for GBS increased at slow strain rates. 

Figure 8a demonstrates that present creep data for high stresses can be reasonably modelled by dynamic recovery of dislocations at HAGBs (Equation (A2)). This model was proposed by Blum et al. [22]. They suggested that HAGBs can accelerate creep strain rate through enhanced recovery of dislocations via grain boundary diffusion. The preferential recovery of the microstructure in the vicinity of prior austenite grain boundaries (PAGBs) was also observed in coarse-grained 9% Cr steels [25,56]. It was suggested that the preferential recovery along PAGBs leads to the significant decrease in creep strength and causes premature failure. The loss of creep strength has also been explained by the formation of the secondary phases, such as the Laves and Z phases. The formation Z phase causes a decrease in the fine carbide and nitride precipitates [57], and its subsequent fast coarsening leads to the loss of creep strength. The formation of the Laves phase decreases solid solution strengthening, and coarse Laves particles can serve as the nucleation sites for cavities [31]. This premature failure was also observed in the present UFG P92 steel (Figure 8b). However, the premature failure occurrence in UFG P92 steel was shifted to a shorter time to failure and lower stresses in comparison with coarse-grained P92 steel (Figure 8b). Due to the short-term of creep testing, it is suggested that the premature failure in UFG P92 steel was not associated with the formation of Z phase. In fact, the formation of the Z phase was not observed in the UFG microstructure. Thus, the premature failure of the UFG P92 steel can probably be associated with the enhanced recovery of dislocation at the HAGBs and the enhanced coarsening of Laves phase. 

The present results indicate that under low stresses, the minimum creep rates measured for UFG P92 steel approach a Nabarro–Herring type diffusion creep mechanism of (Equation (A3)). The occurrence of a creep mechanism with *n* close to 1 and a decrease in the activation energy, *Q*_c_, in coarse-grained 9% Cr steel was found by Kloc et al. [52]. The present work contains only limited creep results measured under low stress. It seems that the shapes of the experimental curves measured for both UFG and CG P92 are likely similar, but the experimental results measured for UFG P92 steel were shifted to faster strain rates and lower stresses in comparison with the CG state.

## 6. Conclusions

- The application of 1 HPT at room temperature deteriorated creep resistance in comparison with the CG state. UFG P92 steel exhibited lower values of the stress exponent *n* in comparison with experimental results published for CG P92 steel tested under at the same creep conditions.
- The microstructure investigation revealed that the formation of the Laves phase could contribute to the strengthening of UFG P92 steel during the primary creep stage.
- The creep results revealed the occurrence of premature failure in creep tests of UFG P92 steel. The premature failure was shifted to shorter times and lower stresses in comparison with coarse-grained P92 steel.
- The comparison of experimental results with predictions from creep models indicates that the creep behaviour of UFG P92 steel could be influenced by enhanced recovery of dislocations at HAGBs and the Nabarro–Hering creep mechanism under low stresses.

## Figures and Tables

**Table 1:** Chemical composition (wt. %) of the investigated P92 pipe.

Element	C	Cr	Mo	W	Si	Mn	V	Nb	P	N	Al	S
wt. %	0.11	8.58	0.33	1.67	0.37	0.48	0.23	0.06	0.013	0.037	0.017	0.005

**Table 2:** The slopes in different regions of the primary creep stage (Figure 2a).

Stress [MPa]	160	150	100	80
Region I slope	0.43	0.53	0.64	0.71
Region II slope	0.64	0.78	0.84	1.41

## Appendix A. Equations Used for Modelling

### Appendix A.1. Grain Boundary Sliding

The creep behaviour can be controlled by grain boundary sliding [58]:

(A1) $${\dot{\varepsilon }}_{m}=\pi \frac{D_{b}Gb}{k_{B}T}{\left(\frac{b}{d}\right)}^{2}{\left(\frac{\sigma }{G}\right)}^{2},$$

where *D_b_* = 7.03 × 10^−14^ (m^2^/s) [59] is the boundary diffusion coefficient, *G* is the shear modulus, *d* is grain size, *b* is the Burgers vector length, and *k_B_* is the Boltzmann constant.

### Appendix A.2. HAGBs Control of Recovery in Pure Materials

The creep behaviour of a fully UFG microstructure controlled by the storage and dynamic recovery of free dislocations at high-angle boundaries is given by following equation [22]:

(A2) $${\dot{\varepsilon }}_{m}=A\frac{D_{b}Gb}{k_{B}T}{\left(\frac{d}{b}\right)}^{4}{\left(\frac{\sigma }{G}\right)}^{8}.$$

The numerical constant A was calculated by setting the four fit parameters, *f*_1_, in [22,60] to 0.3 and the dislocation interaction factor α to 0.25. In contrast to Equation (A1), Equation (A3) predicts an increase of $\dot{\varepsilon }$ with an increase in *d*.

### Appendix A.3. Nabarro–Hering Diffusion Creep

The formula for the diffusion of Nabarro–Hering creep in the stationary state of deformation follows [61]:

(A3) $${\dot{\varepsilon }}_{m}=A_{NH}\frac{D_{L}Gb}{k_{B}T}{\left(\frac{b}{d}\right)}^{2}{\left(\frac{\sigma }{G}\right)}^{1},$$

where *D_L_* = 1.58 × 10^−19^ (m^2^/s) [62] is the lattice diffusion coefficient, and *A_NH_* is a constant is equal to 2 [63].
